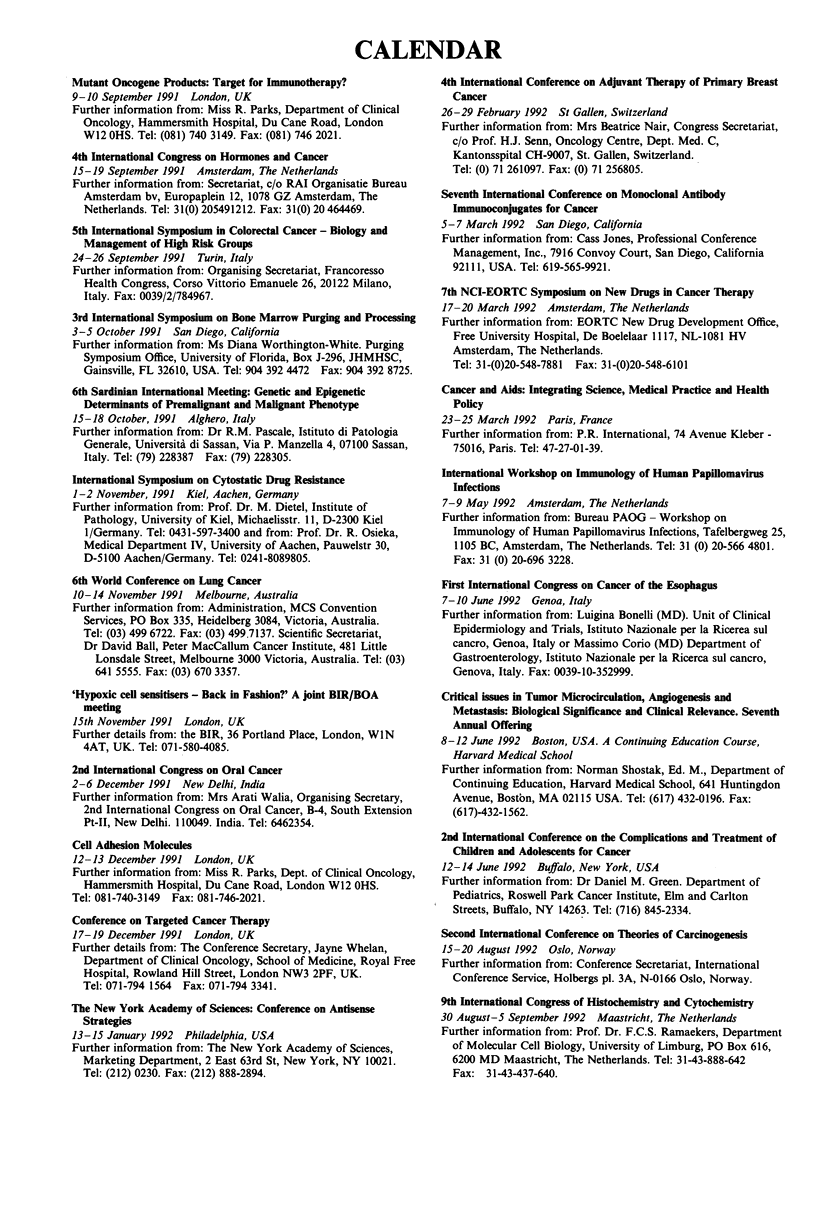# Calendar

**Published:** 1991-10

**Authors:** 


### CALENDAR

**Mutant Oncogene Products: Target for Immunotherapy?**
*9–10 September 1991 London, UK*
Further information from: Miss R. Parks, Department of Clinical Oncology, Hammersmith Hospital, Du Cane Road, London W12 0HS. Tel: (081) 740 3149. Fax: (081) 746 2021.

**4th International Congress on Hormones and Cancer**
*15–19 September 1991 Amsterdam, The Netherlands*
Further information from: Secretariat, c/o RAI Organisatie Bureau Amsterdam bv, Europaplein 12, 1078 GZ Amsterdam, The Netherlands. Tel: 31(0) 205491212. Fax: 31(0) 20 464469.

**5th International Symposium in Colorectal Cancer – Biology and Management of High Risk Groups**
*24–26 September 1991 Turin, Italy*
Further information from: Organising Secretariat, Francoresso Health Congress, Corso Vittorio Emanuele 26, 20122 Milano, Italy. Fax: 0039/2/784967.

**3rd International Symposium on Bone Marrow Purging and Processing**
*3–5 October 1991 San Diego, California*
Further information from: Ms Diana Worthington-White. Purging Symposium Office, University of Florida, Box J-296, JHMHSC, Gainsville, FL 32610, USA. Tel: 904 392 4472 Fax: 904 392 8725.

**6th Sardinian International Meeting: Genetic and Epigenetic Determinants of Premalignant and Malignant Phenotype**
*15–18 October, 1991 Alghero, Italy*
Further information from: Dr R.M. Pascale, Istituto di Patologia Generale, Università di Sassan, Via P. Manzella 4, 07100 Sassan, Italy. Tel: (79) 228387 Fax: (79) 228305.

**International Symposium on Cytostatic Drug Resistance**
*1–2 November, 1991 Kiel, Aachen, Germany*
Further information from: Prof. Dr. M. Dietel, Institute of Pathology, University of Kiel, Michaelisstr. 11, D-2300 Kiel 1/Germany. Tel: 0431-597-3400 and from: Prof. Dr. R. Osieka, Medical Department IV, University of Aachen, Pauwelstr 30, D-5100 Aachen/Germany. Tel: 0241-8089805.

**6th World Conference on Lung Cancer**
*10–14 November 1991 Melbourne, Australia*
Further information from: Administration, MCS Convention Services, PO Box 335, Heidelberg 3084, Victoria, Australia. Tel: (03) 499 6722. Fax: (03) 499 7137. Scientific Secretariat, Dr David Ball, Peter MacCallum Cancer Institute, 481 Little Lonsdale Street, Melbourne 3000 Victoria, Australia. Tel: (03) 641 5555. Fax: (03) 670 3357.

**'Hypoxic cell sensitisers – Back in Fashion?' A joint BIR/BOA meeting**
*15th November 1991 London, UK*
Further details from: the BIR, 36 Portland Place, London, W1N 4AT, UK. Tel: 071-580-4085.

**2nd International Congress on Oral Cancer**
*2–6 December 1991 New Delhi, India*
Further information from: Mrs Arati Walia, Organising Secretary, 2nd International Congress on Oral Cancer, B-4, South Extension Pt-II, New Delhi. 110049. India. Tel: 6462354.

**Cell Adhesion Molecules**
*12–13 December 1991 London, UK*
Further information from: Miss R. Parks, Dept. of Clinical Oncology, Hammersmith Hospital, Du Cane Road, London W12 0HS.
Tel: 081-740-3149 Fax: 081-746-2021.

**Conference on Targeted Cancer Therapy**
*17–19 December 1991 London, UK*
Further details from: The Conference Secretary, Jayne Whelan, Department of Clinical Oncology, School of Medicine, Royal Free Hospital, Rowland Hill Street, London NW3 2PF, UK.
Tel: 071-794 1564 Fax: 071-794 3341.

**The New York Academy of Sciences: Conference on Antisense Strategies**
*13–15 January 1992 Philadelphia, USA*
Further information from: The New York Academy of Sciences, Marketing Department, 2 East 63rd St, New York, NY 10021. Tel: (212) 0230. Fax: (212) 888-2894.

**4th International Conference on Adjuvant Therapy of Primary Breast Cancer**
*26–29 February 1992 St Gallen, Switzerland*
Further information from: Mrs Beatrice Nair, Congress Secretariat, c/o Prof. H.J. Senn, Oncology Centre, Dept. Med. C, Kantonsspital CH-9007, St. Gallen, Switzerland.
Tel: (0) 71 261097. Fax: (0) 71 256805.

**Seventh International Conference on Monoclonal Antibody Immunoconjugates for Cancer**
*5–7 March 1992 San Diego, California*
Further information from: Cass Jones, Professional Conference Management, Inc., 7916 Convoy Court, San Diego, California 92111, USA. Tel: 619-565-9921.

**7th NCI-EORTC Symposium on New Drugs in Cancer Therapy**
*17–20 March 1992 Amsterdam, The Netherlands*
Further information from: EORTC New Drug Development Office, Free University Hospital, De Boelelaar 1117, NL-1081 HV Amsterdam, The Netherlands.
Tel: 31-(0)20-548-7881 Fax: 31-(0)20-548-6101

**Cancer and Aids: Integrating Science, Medical Practice and Health Policy**
*23–25 March 1992 Paris, France*
Further information from: P.R. International, 74 Avenue Kleber - 75016, Paris. Tel: 47-27-01-39.

**International Workshop on Immunology of Human Papillomavirus Infections**
*7–9 May 1992 Amsterdam, The Netherlands*
Further information from: Bureau PAOG – Workshop on Immunology of Human Papillomavirus Infections, Tafelbergweg 25, 1105 BC, Amsterdam, The Netherlands. Tel: 31 (0) 20-566 4801. Fax: 31 (0) 20-696 3228.

**First International Congress on Cancer of the Esophagus**
*7–10 June 1992 Genoa, Italy*
Further information from: Luigina Bonelli (MD). Unit of Clinical Epidermiology and Trials, Istituto Nazionale per la Ricerea sul cancro, Genoa, Italy or Massimo Corio (MD) Department of Gastroenterology, Istituto Nazionale per la Ricerca sul cancro, Genova, Italy. Fax: 0039-10-352999.

**Critical issues in Tumor Microcirculation, Angiogenesis and Metastasis: Biological Significance and Clinical Relevance. Seventh Annual Offering**
*8–12 June 1992 Boston, USA. A Continuing Education Course, Harvard Medical School*
Further information from: Norman Shostak, Ed. M., Department of Continuing Education, Harvard Medical School, 641 Huntingdon Avenue, Boston, MA 02115 USA. Tel: (617) 432-0196. Fax: (617)-432-1562.

**2nd International Conference on the Complications and Treatment of Children and Adolescents for Cancer**
*12–14 June 1992 Buffalo, New York, USA*
Further information from: Dr Daniel M. Green. Department of Pediatrics, Roswell Park Cancer Institute, Elm and Carlton Streets, Buffalo, NY 14263. Tel: (716) 845-2334.

**Second International Conference on Theories of Carcinogenesis**
*15–20 August 1992 Oslo, Norway*
Further information from: Conference Secretariat, International Conference Service, Holbergs pl. 3A, N-0166 Oslo, Norway.

**9th International Congress of Histochemistry and Cytochemistry**
*30 August–5 September 1992 Maastricht, The Netherlands*
Further information from: Prof. Dr. F.C.S. Ramaekers, Department of Molecular Cell Biology, University of Limburg, PO Box 616, 6200 MD Maastricht, The Netherlands. Tel: 31-43-888-642
Fax: 31-43-437-640.